# Supplementary figures and images for: Methylene blue inhibits nucleation and elongation of SOD1 amyloid fibrils
Source: PeerJ. 2020 Aug 14;8:e9719. doi: 10.7717/peerj.9719 (PMC7430317; doi:10.7717/peerj.9719)

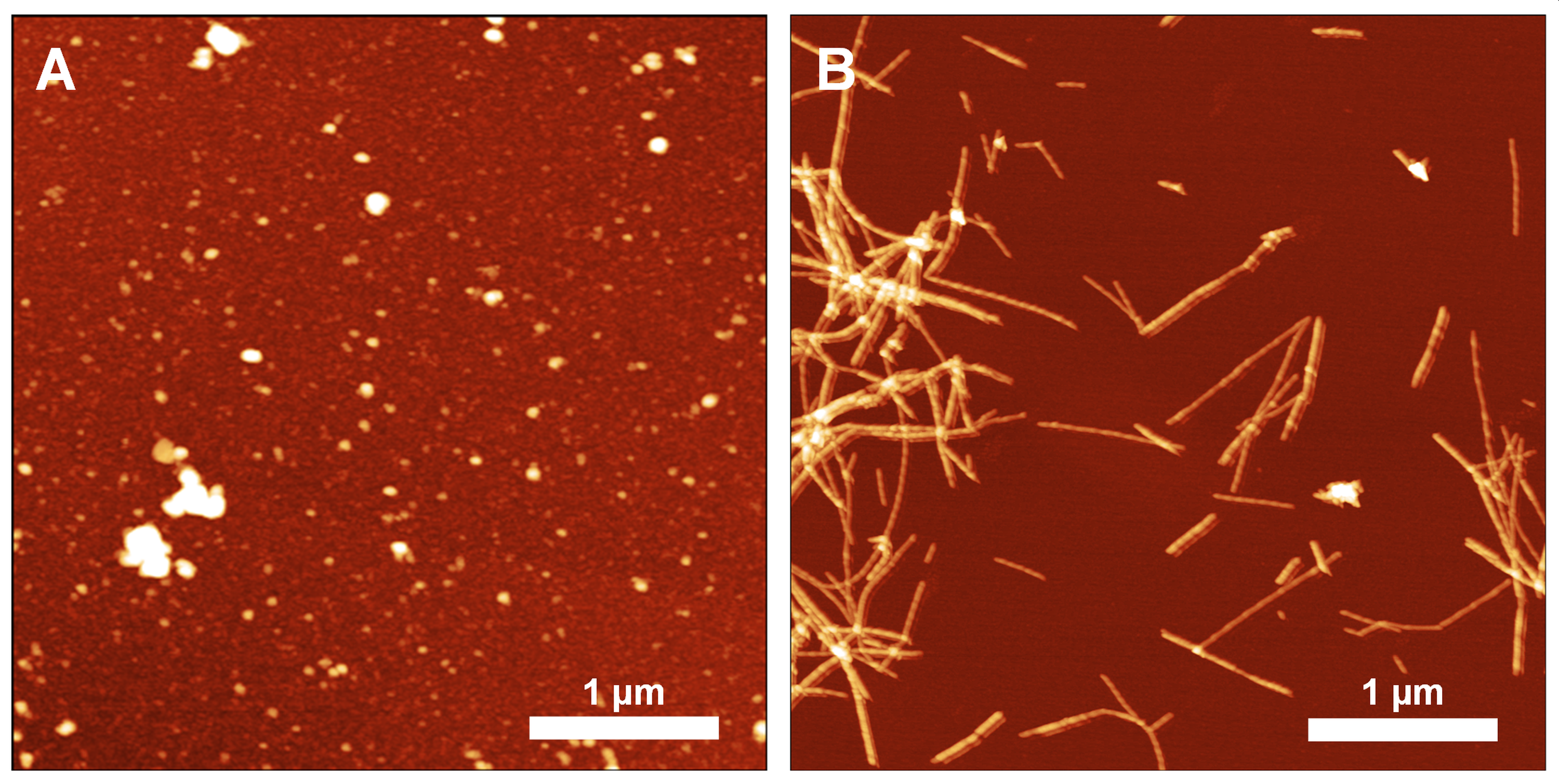

Supplement: Supplemental Information 1 — Formation of round-shaped oligomers in the absence of GuHCl (A) and thread-like amyloid fibers in the presence of 0.5 M GuHCl (B). [file peerj-08-9719-s001.png]

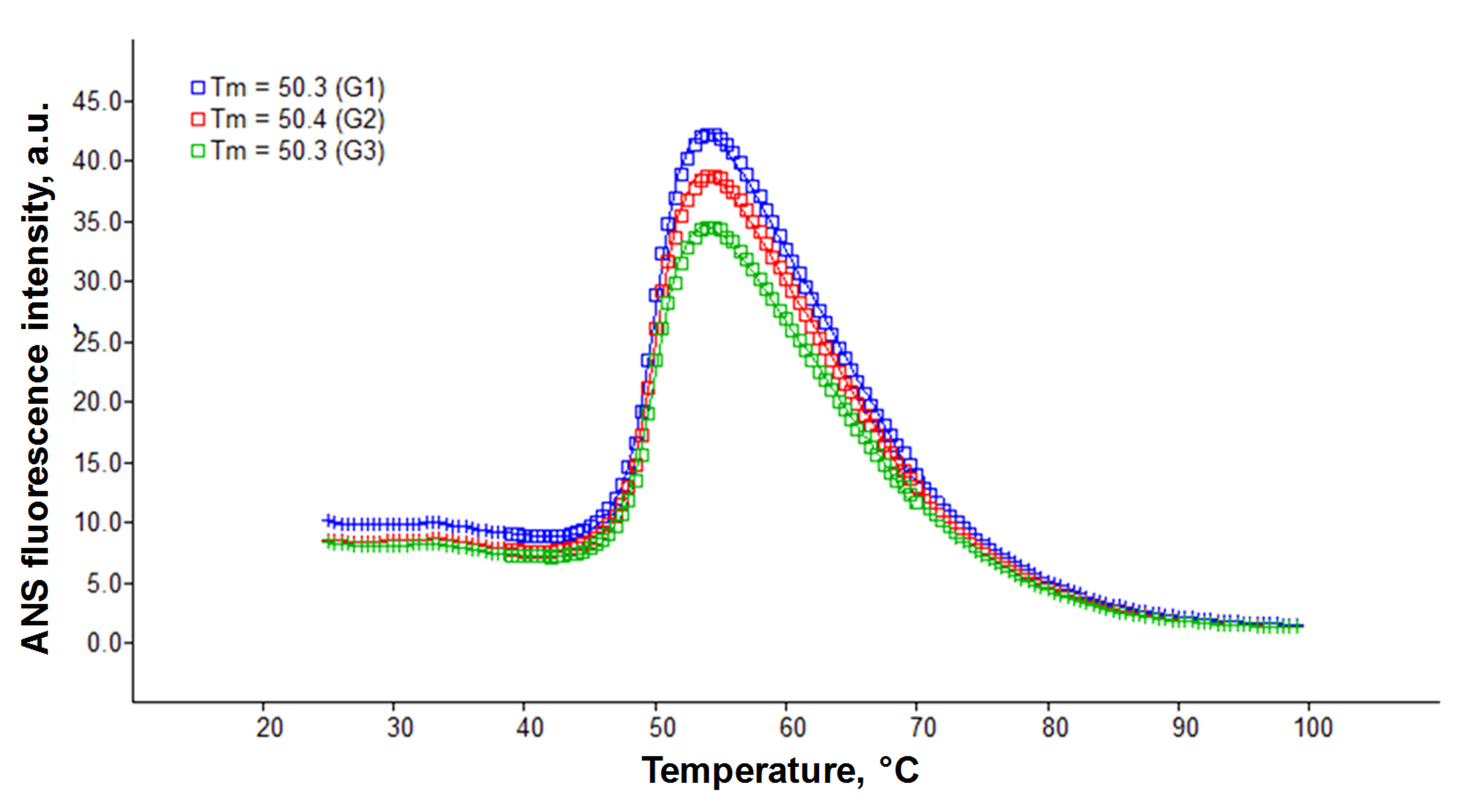

Supplement: Supplemental Information 2 — Three repeats of ANS fluorescence intensity dependencies on temperature. [file peerj-08-9719-s002.png]

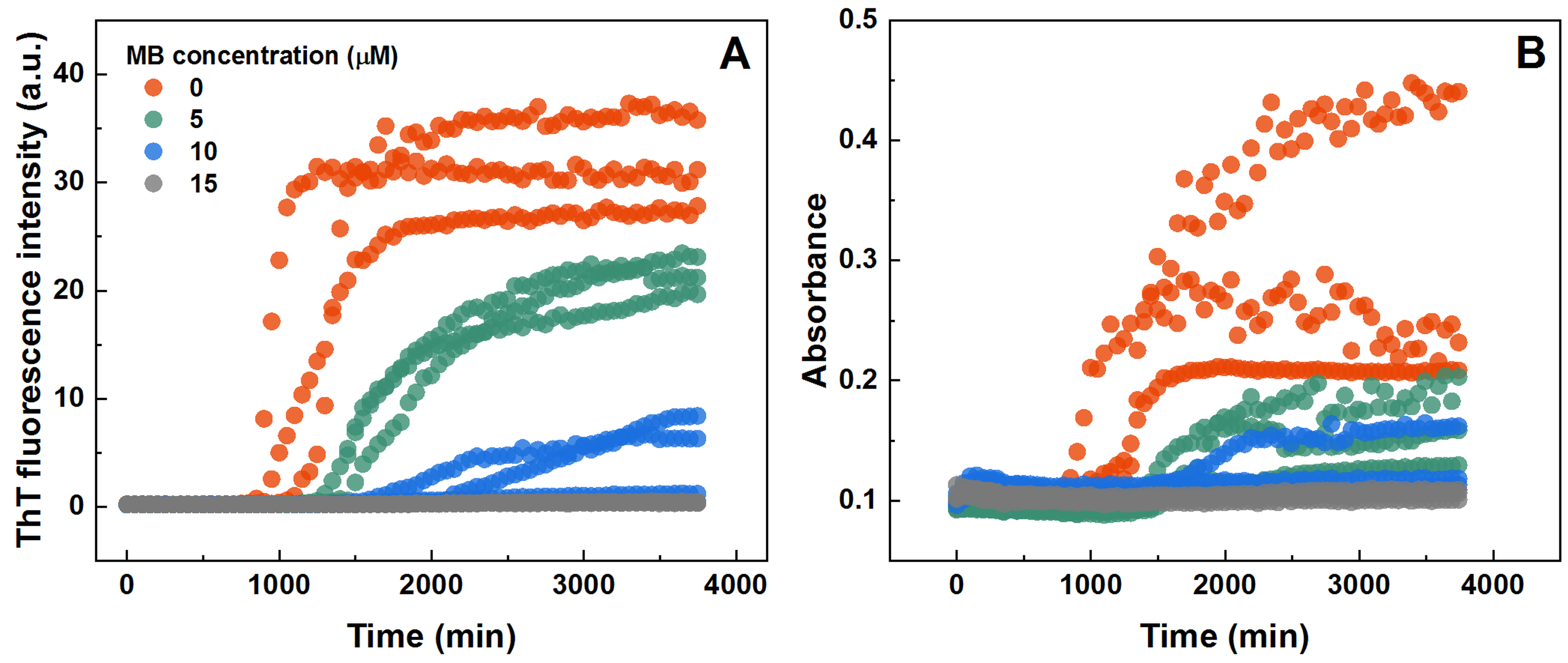

Supplement: Supplemental Information 3 — SOD1 aggregated in 10 mM potassium phosphate buffer with 0.5 M GuHCl and 5 mM DTT with 0–15 µM MB, pH 7.4. [file peerj-08-9719-s003.png]

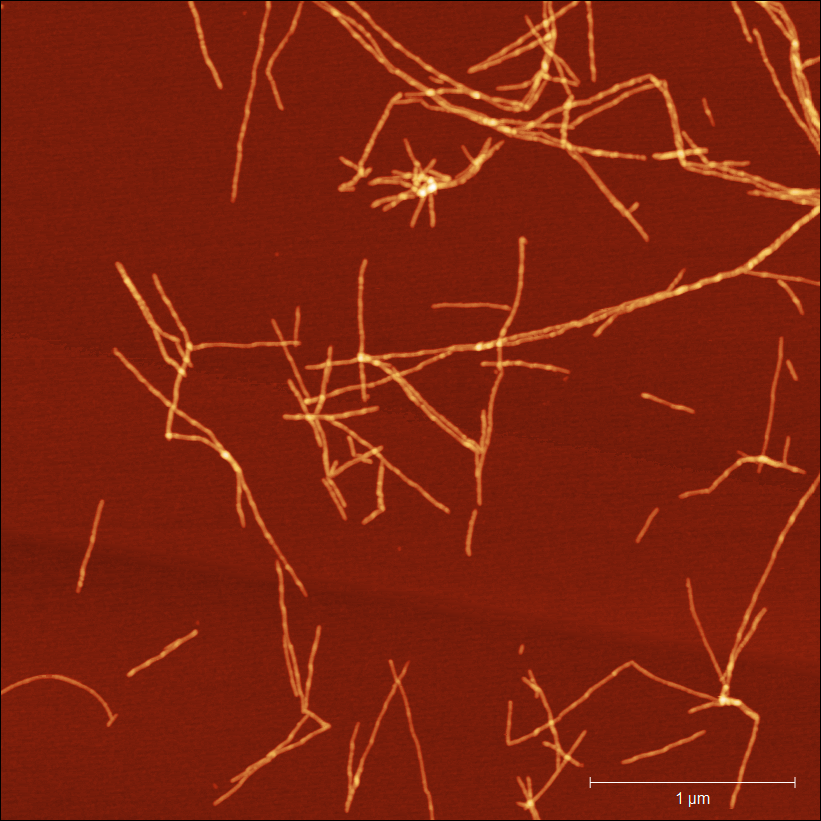

Supplement: Supplemental Information 5 [file peerj-08-9719-s005.zip › Fig 3B.tiff]

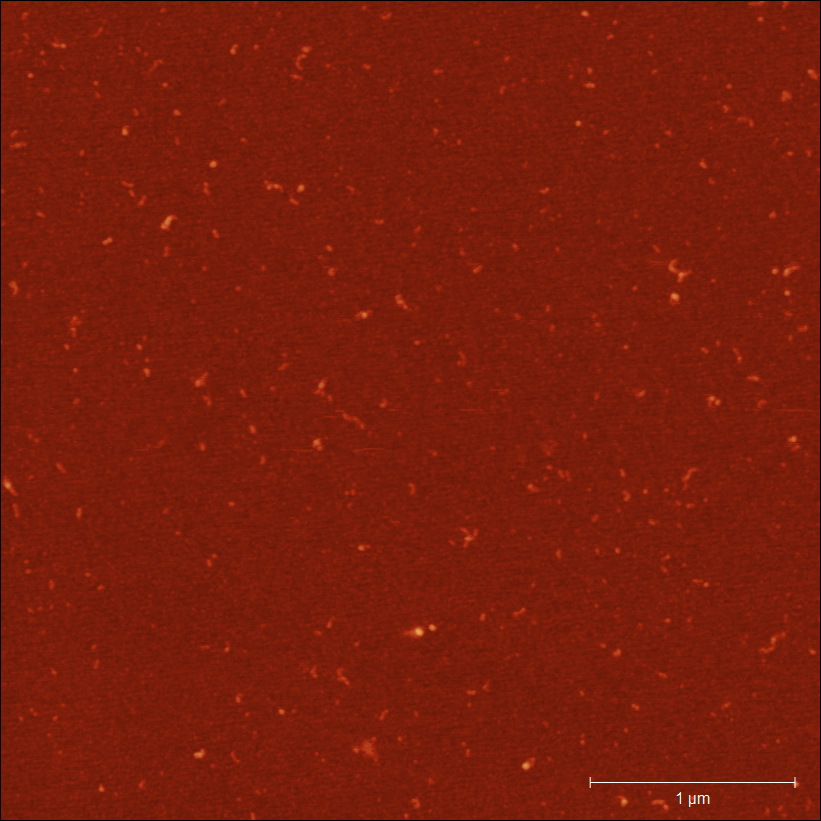

Supplement: Supplemental Information 5 [file peerj-08-9719-s005.zip › Fig 3C.tiff]

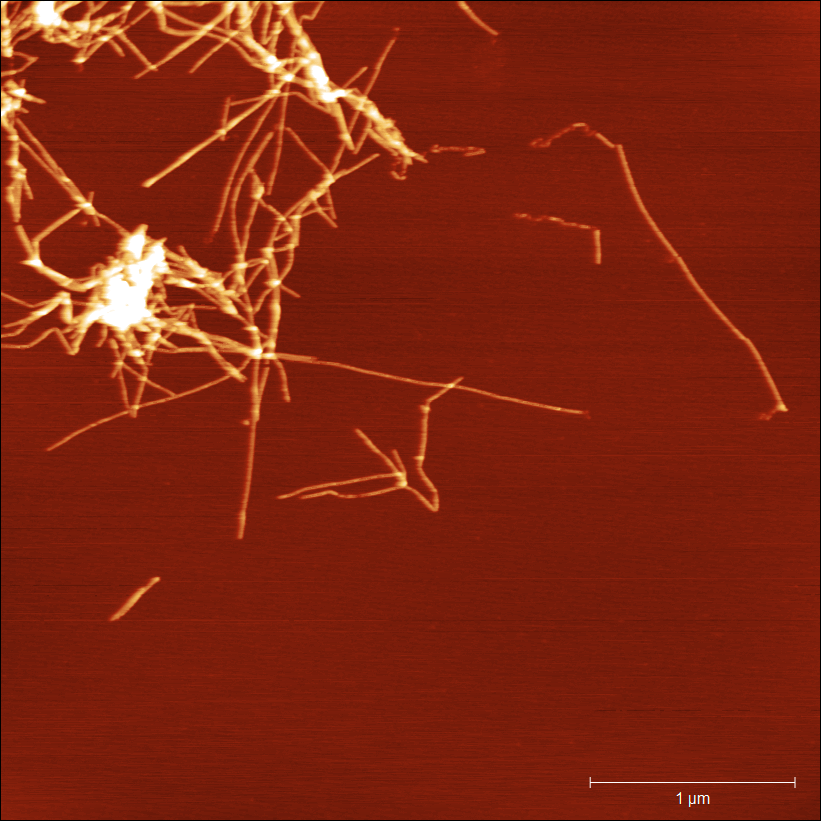

Supplement: Supplemental Information 5 [file peerj-08-9719-s005.zip › Fig 4A.tiff]

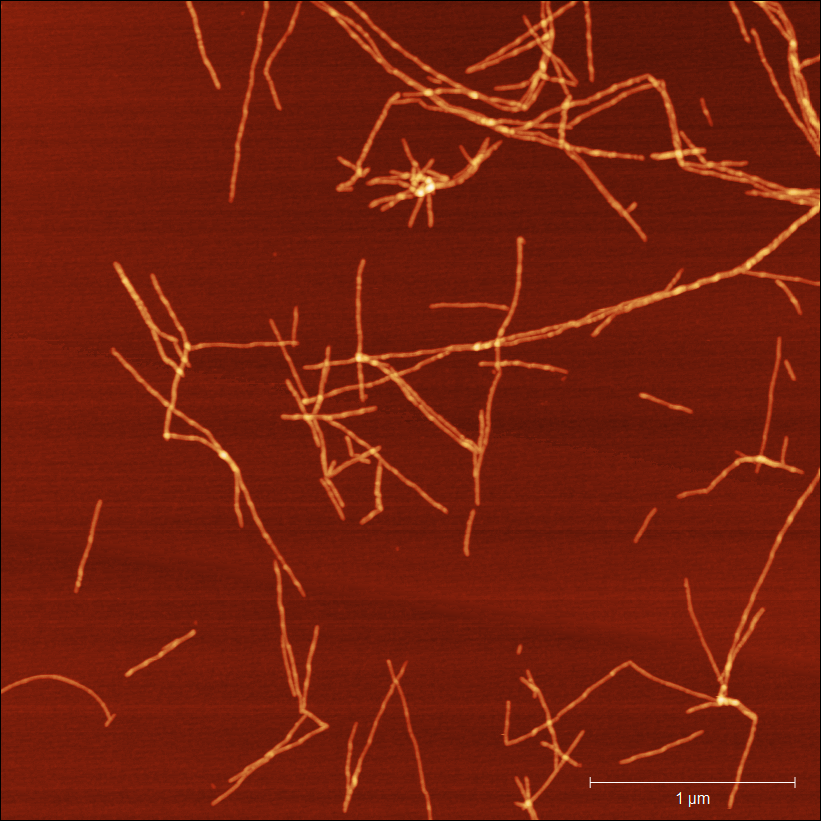

Supplement: Supplemental Information 5 [file peerj-08-9719-s005.zip › Fig 5D.tiff]

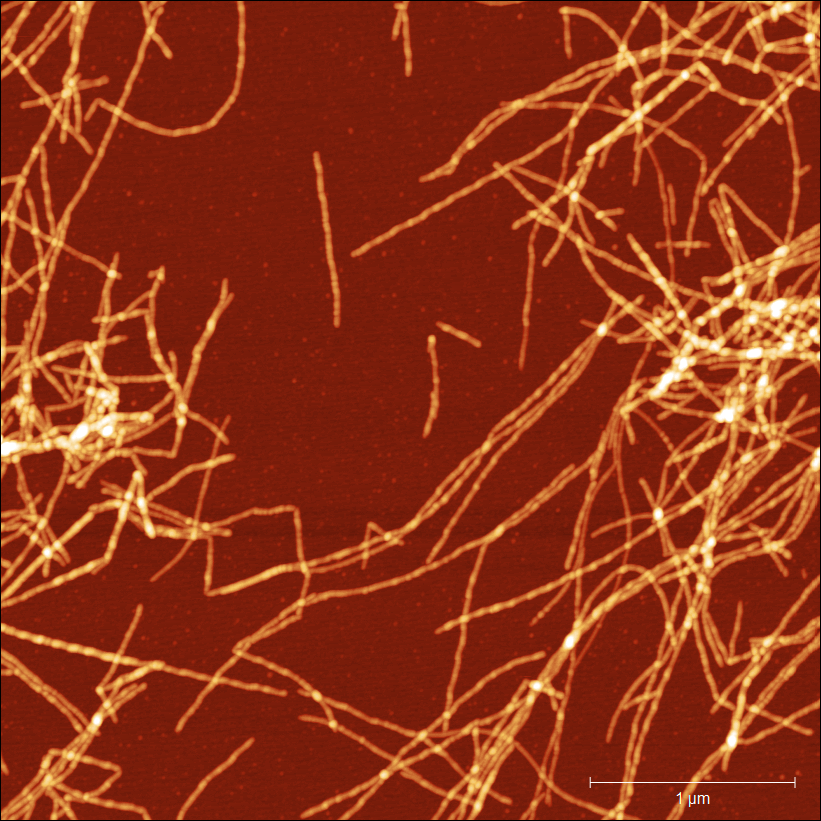

Supplement: Supplemental Information 5 [file peerj-08-9719-s005.zip › Fig 5E.tiff]

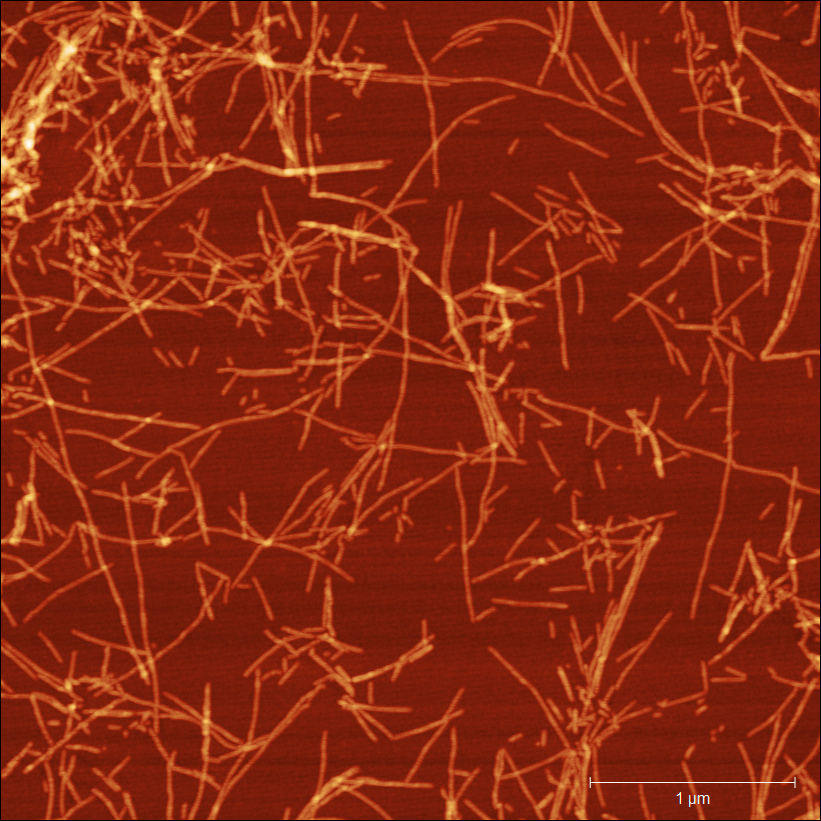

Supplement: Supplemental Information 5 [file peerj-08-9719-s005.zip › Fig 1B.tiff]

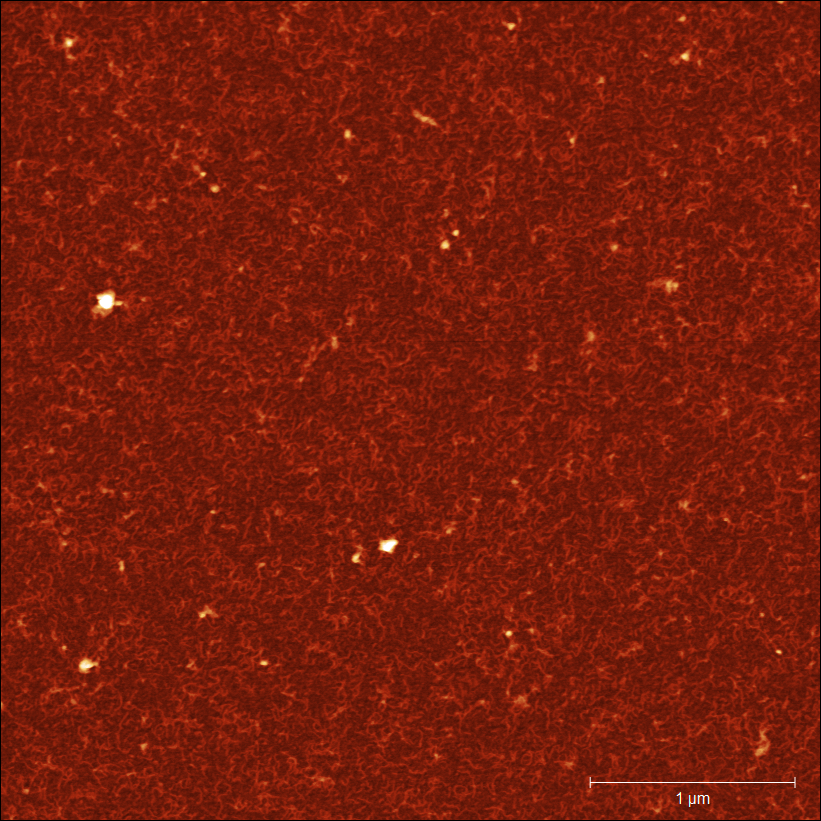

Supplement: Supplemental Information 5 [file peerj-08-9719-s005.zip › Fig 1C.tiff]

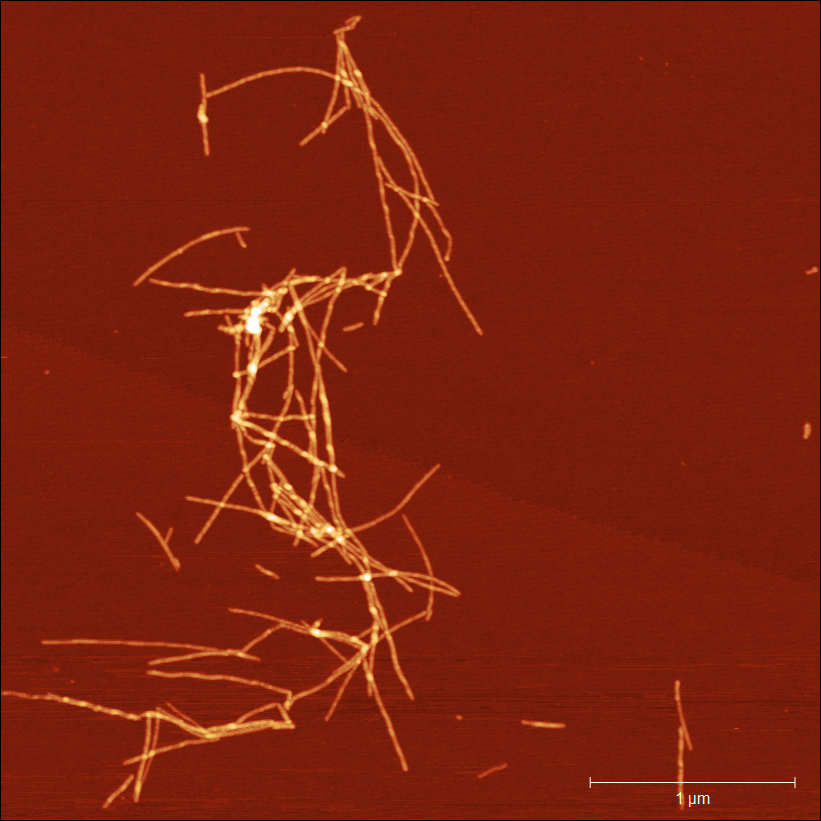

Supplement: Supplemental Information 5 [file peerj-08-9719-s005.zip › Fig 2G.tiff]

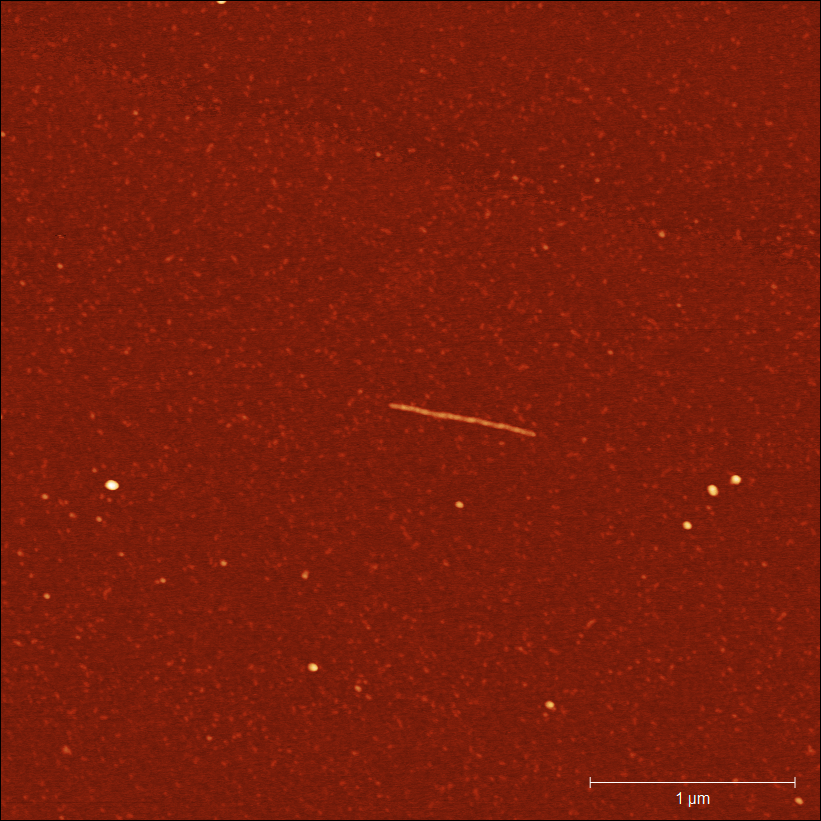

Supplement: Supplemental Information 5 [file peerj-08-9719-s005.zip › Fig 2H.tiff]
